# Atopic dermatitis, HSV and IgE: clinical and laboratory relationship

**DOI:** 10.1186/1939-4551-8-S1-A55

**Published:** 2015-04-08

**Authors:** Amanda Seba

**Affiliations:** 1 Clementino Fraga Filho Hospital, Federal University of Rio De Janeiro, Brazil

## Background

Atopic dermatitis (AD) is a chronic inflammatory dermatosis of the skin that leads to xerosis and pruritus. It is known that the serum levels of total immunoglobulin E (IgE) appear to be higher in patients with more severe AD. Changes in skin barrier also demonstrate that there is increased susceptibility to skin infections in AD, including by viruses, such as herpes simplex virus (HSV).

## Methods

The study was retrospective and cross-sectional, with chart review. Patients were selected for monitoring in the immunology service, aged between 16 and 75 years with diagnosis of AD by Hanifin and Rajka criteria updated in 2003, classified as mild, moderate or severe AD by calculating the EASI (*Eczema Area and Severity Index*), which had HSV serology and total IgE. Serology was performed by ELISA (*enzyme linked immunosorbent assay*) and serum total IgE by nephelometry with N Latex IgE mono ®.

## Results

Twenty-four patients participated in the study. Of these, 14 women, 13 white, 20 seropositive for HSV, six with severeAD, 12 moderate and 6 severe. The total IgE values were variable, with the highest observed in more severe patients. Weana lyzed the clinical and laboratory relationship between serum levels of total IgE and positive serology for HSV. This was not statist ically significant relationship. Patients with highest total levels of IgE were not more likely to express HSV infection, with worst prognosis of AD.

## Conclusions

There was no relationship between HSV soropositivity, elevated IgE serum levels and increased severity of AD.

